# Fabrication of Straight Silicon Nanowires and Their Conductive Properties

**DOI:** 10.1186/s11671-015-1025-x

**Published:** 2015-08-14

**Authors:** S. Wu, Y. M. Shao, T. X. Nie, L. Xu, Z. M. Jiang, X. J. Yang

**Affiliations:** State Key Laboratory of Surface Physics and Collaborative Innovation Center of Advanced Microstructures, Fudan University, Shanghai, 200433 China; Department of Electrical Engineering, University of California, Los Angeles, CA 90095 USA

**Keywords:** Si nanowires, Annealing conditions, Properties, Conductive atomic force microscopy

## Abstract

Straight Si nanowires (Si NWs) with tens to hundreds of micrometers in length and 40–200 nm in diameter are achieved by annealing a Si substrate coated with metallic Fe. The influences of annealing gas and temperature on the formation of Si NWs are investigated. It is found that the annealing gas has significant impacts on the microstructure of the NWs. By increasing the hydrogen ratio in the forming gas, straight and crystal Si NWs with thin oxide shells are obtained. Both the conductive properties along and perpendicular to the NW are investigated by conductive atomic force microscopy (CAFM) on single NWs. The conductance perpendicular to the NW is too poor to be detected, while a weak conductance can be measured along the NW. The results indicate that the measured resistance mainly comes from the contact(s), and the Si NWs exhibit typical semiconductive conductance themselves, which should have potential applications in nanoelectronics.

## Background

Silicon nanowires (Si NWs) have many unique electronic, optoelectronic, and mechanical properties owing to their anisotropic morphology, high surface-to-volume ratio, tailorable bandgap, and quantum confinement [1, 2]. Due to these unique properties and compatibility with traditional silicon technology, Si NWs have been demonstrated for a variety of applications, such as field-effect transistors, solar cells, integrated logic circuits, lithium-ion batteries, thermoelectric devices, biosensors, and many others [3–10]. In the past decades, considerable efforts have been devoted to nanowire synthesis and a variety of methods have been established for growing Si and its oxide NWs [7–19]. Among these growth methods, solid-liquid-solid (SLS) growth is a relatively straightforward technique for producing Si NWs [11, 12], because in SLS the silicon substrate itself serves as the silicon source, with no additional Si source needed like that in vapor-liquid-solid (VLS) growth [13, 14]. However, the SLS-grown NWs are usually in a mess of curved wires and typically have thick oxide shells or incorporate nonstoichiometric amounts of oxygen [11, 12, 15–19]. Therefore, the growth of straight and crystal Si NWs with thin or no oxide shell is still a challenge and well demanded for nanoelectronic applications.

On the other hand, in order to realize the applications, it is extremely important to get a good understanding of their electrical properties. However, compared to the intensive studies on the growth of NWs, studies on their electrical property are few, especially on single Si NWs. Scanning probe microscopy (SPM)-based electrical measurements reveal themselves as powerful techniques for electrical characterizations at nanoscale [20, 21]. Among these SPM techniques, conductive atomic force microscopy (CAFM) has been most often applied to study the conductive properties of individual nanostructures such as films, heterostructures, dots, or nanoparticles [22–25]. The CAFM studies on one-dimensional nanostructures have also been attempted, including GaN [26], CdTe [27], FeSi_2_ [28], CuO [29] and Bi_2_S_3_ [30] NWs, ZnO nanoneedles [31], and carbon nanotubes [32, 33]. There are also a few studies reported for Si NWs, such as the conductance measurements on horizontal and vertical NWs prepared by different methods [34], and the dielectric property studies on single Si nanowire oxide [35]. Despite all of these efforts, the CAFM studies on single NWs still need to be improved. It is mainly due to the electrical contact problem between the NW and the tip as well as that between the NW and substrate (or electrode) [36], and/or the easy distortion or damage of the NW by the CAFM tip, particularly for the NWs separated from the substrate.

In this letter, we demonstrate an approach to synthesize straight Si NWs with thin oxide shells and crystal structures by increasing the hydrogen ratio in the annealing gas. Their conductive properties perpendicular to and along the NWs are investigated by CAFM on single NWs. Due to the poor electrical contact(s), the conductance of Si NWs perpendicular to the NW cannot be detected, whereas a weak conductance is measured along the NW with the contact between the NW and the electrode improved.

## Methods

P-type Si (100) wafers with a resistivity of 5~10 Ω cm were chemically cleaned with the Shiraki method [37] and then loaded into the ultrahigh vacuum growth chamber of the molecular beam epitaxy (MBE) system. After the thermal treatment at 950 °C for 10 min to remove the protected silicon dioxide layer, a 1-nm-thick Fe film was deposited on the Si wafer at room temperature by MBE. The choice of Fe as catalyst is because Fe would not form a solid solution with Si according to the Si-Fe phase diagram, so that the possibility for Fe contaminating Si NWs can be avoided [38]. An annealing furnace equipped with a temperature controller and a quartz tube was used for the NW synthesis. Si NWs were synthesized at different annealing temperatures in forming gas (with a flow rate of 150 L/h) at atmospheric pressure. Before annealing, the forming gas was flowed through the tube for more than 20 min to expel out the oxygen gas in the tube. After the completion of the NW synthesis, the furnace was cooled naturally to room temperature in flowing forming gas.

**Fig. 1:**
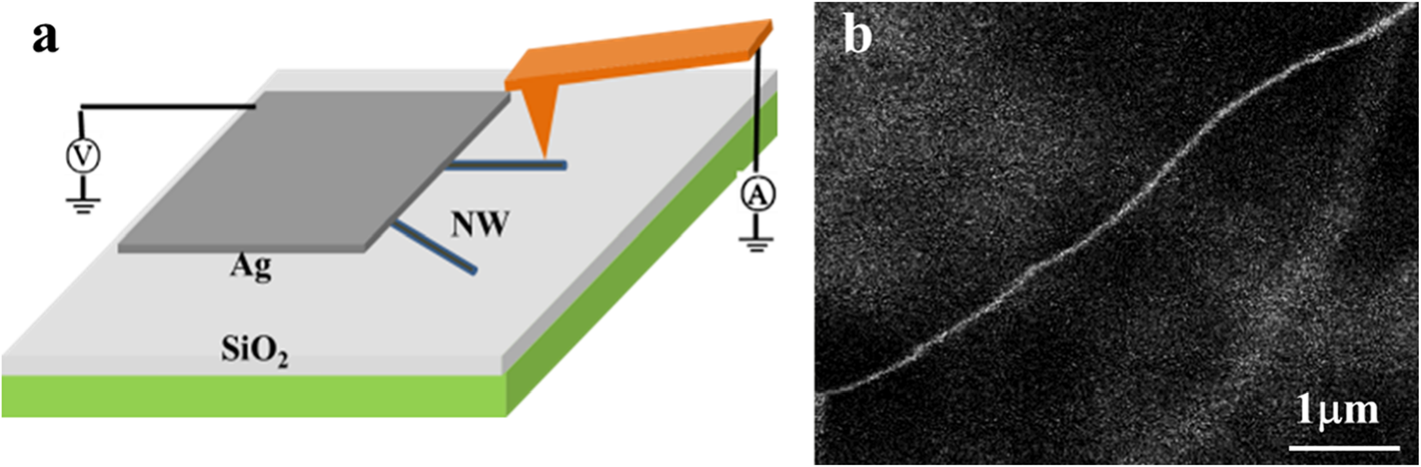
**a** The diagram showing the conductance measurements along the NW by CAFM. **b** SEM image of a single Si NW spread on a Si substrate, which is formed by annealing in 10 % H_2_ forming gas at 1250 °C

The morphological and nanostructural characteristics of the synthesized products were measured by scanning electron microscopy (SEM), transmission electron microscopy (TEM), and Raman spectroscopy, and the conductive properties were investigated by CAFM. To prepare the samples for the nanostructural and electrical measurements, Si NWs were scraped off the Si substrate, and then the scraped powers were ultrasonically dispersed in ethanol. A drop of solution was deposited on a Cu mesh or Ta substrate for TEM and Raman measurements, respectively. To prepare the samples for CAFM measurements, the nanowires were dipped in 10 % HF solution for 2 min to remove the oxide layer before being scraped off the substrate and dispersed in ethanol immediately after scraping. The ethanol-dispersed solution was dropped on the chemically cleaned p-type Si substrate (with oxide removed by HF etching) for the measurements perpendicular to nanowires, or on the SiO_2_-covered Si substrate for the measurements along the nanowires, respectively. To do the latter electrical measurements, one side of the Si NWs was buried under the Ag electrode which was prepared with silver epoxy, as schemed in Fig. 1, together with the SEM image of a single scattered Si NW on the Si substrate. The conductive properties of single Si NWs were investigated by CAFM in contact mode by applying a DC voltage between the tip and the sample with a Pt-Ir-coated Si tip. The typical force used for current imaging was about 50 nN, because large forces would easily scrape the NWs off the substrate. In *I*-*V* characteristic measurements, the applied force was also set as 50 nN, since no obvious improvement was achieved under larger forces and instead in some cases NWs would be destroyed. All the electrical experiments were carried out in a flowing nitrogen atmosphere at room temperature.

## Results and Discussion

### Fabrication of Si NWs

**Fig. 2:**
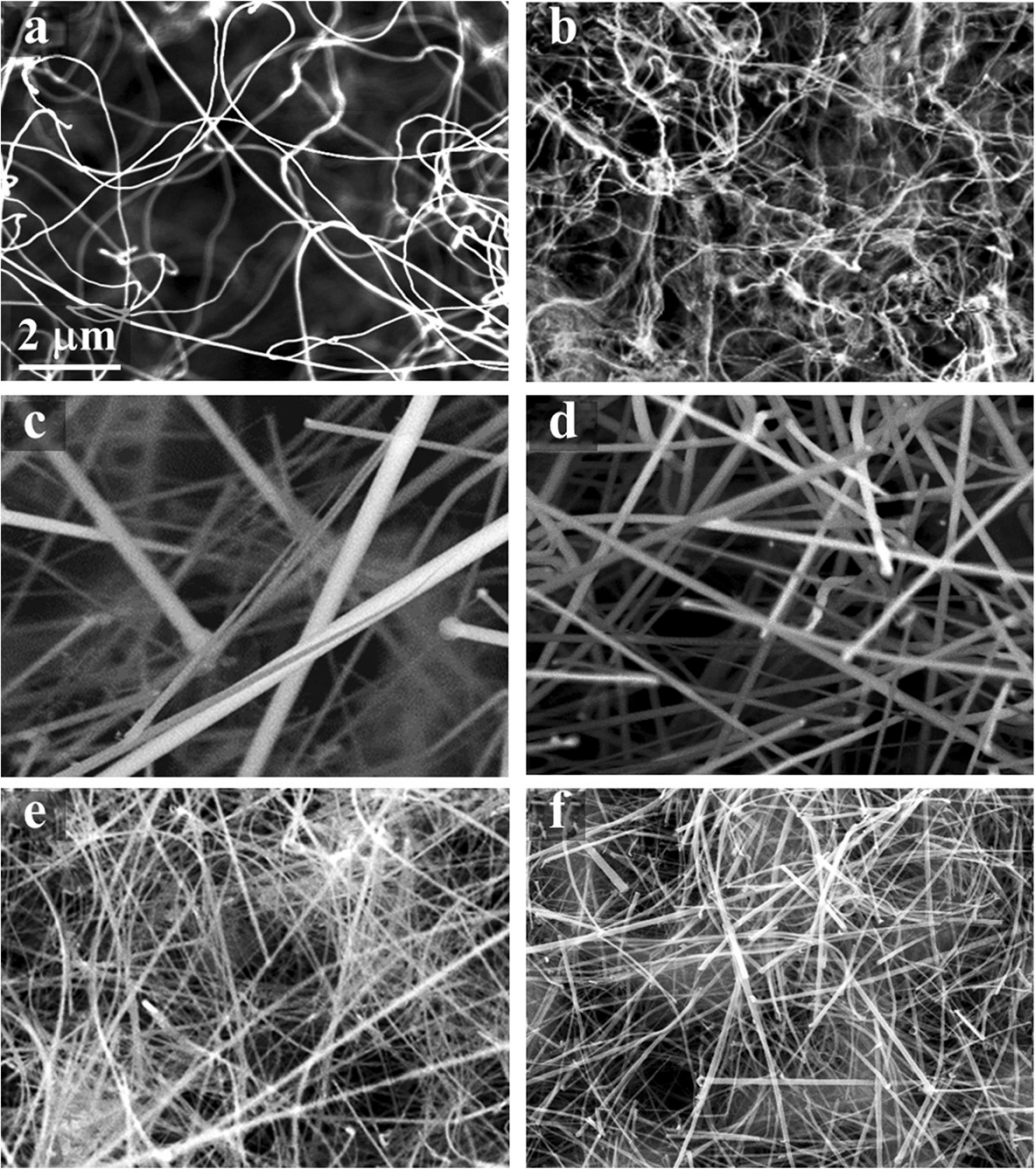
SEM images of Si NW growth at different temperatures in different annealing gases. **a**, **b** Si NWs formed in 5 % H_2_ forming gas at 1200 and 1250 °C, respectively. The NWs annealed in 10 % H_2_ forming gas at **c** 1200 °C, **d** 1250 °C, and **e** 1300 °C. **f** The NWs in **e** after 2 min HF etching

The Si NWs are fabricated by annealing the Si wafer deposited with a thin Fe film in forming gas at high temperatures. The influence of annealing conditions on the formed nanowires is investigated. It is found that the hydrogen ratio in the forming gas has a significant impact on the formed NWs. The topography images of the NWs formed in the forming gas with 5 % H_2_ and 95 % N_2_ (termed as 5 % H_2_ forming gas afterwards) at 1200 °C and 1250 °C are shown in Fig. 2a, b, respectively. Long but curved NWs are observed for both samples, which are about tens to hundreds of micrometers in length and about 40~200 nm in diameter. The averaged diameter of the NWs does not change obviously as the annealing temperature increases from 1200 to 1250 °C, whereas the NWs’ density increases significantly. The results are well consistent with the results reported in our previous paper [12] as well as in other literatures [11, 15–19] with the same SLS approach by applying different catalysts and annealing gases.

On the other hand, much more straight NWs are observed on all the samples annealed in the forming gas with the hydrogen ratio increased to 10 % (termed as 10 % H_2_ forming gas). The topography images of the NWs fabricated at different temperatures of 1200, 1250, and 1300 °C are shown in Fig. 2c–e, respectively. At the annealing temperature of 1200 °C, straight Si NWs with the length up to several tens of micrometers are formed with their diameters largely scattered from 50 to 500 nm. When the annealing temperature increases, the density of the NWs increases, whereas the average diameter of the NWs decreases. Meanwhile, the fluctuation of NWs’ diameter also decreases, which is about 40~200 nm and 30~150 nm for 1250 and 1300 °C annealed samples, respectively. At the annealing temperature of 1300 °C, much dense, long and relatively fine and uniform NWs are formed (Fig. 2e), compared to those annealed at 1200 and 1250 °C, except the NWs are a little curved due to the decreased diameter. From the above results, it can be demonstrated that the annealing gas primary determines the NWs’ shape, while the annealing temperature mainly changes the NWs’ diameter, length, and density.

To check the oxidation status of the NWs, SEM images before and after HF etching are measured. The results found that the NWs annealed in 5 % H_2_ forming gas have little left after HF etching (not shown here), indicating the formation of a thick oxide shell. It is well consistent with the TEM results reported in our previous paper [12], which found that the NWs formed in 5 % H_2_ forming gas exhibited a core-shell structure with a thin Si core (5~7 nm) and a thick Si oxide shell (~40 nm). On the contrary, for the NWs formed by annealing in the 10 % H_2_ forming gas, the etching result is significantly different. Figure 2f presents the SEM image of the NWs as shown in Fig. 2e after a 10 % HF etching for 2 min, and no obvious decrease of the NWs’ diameter is observed. Due to the large scattering of the NWs’ diameter, the exact oxide thickness cannot be deduced from the SEM images, but it can still be declared that the decrease of the NWs’ diameter after HF is small, indicating that these NWs have very thin oxide shells compared to their silicon cores. So, the above results suggest that the annealing gas has a great impact on the formed NWs that would not only change the NWs’ shape, but also vary their chemical constitution.

The reasons why 10 % H_2_ forming gas can greatly inhibit the formation of oxide shell and why straight NWs can be obtained in this case are not very clear at present. Here only a rough explanation is supposed. Due to the contamination, the oxygen in the annealing gas and/or in the annealing tube would react with Fe to form Fe oxide, and the latter will catalyze Si to form SiO_x_ shell in the presence of oxygen contamination, as reported in previous literatures [11, 12]. As the hydrogen ratio in the annealing gas is increased to 10 %, the formation of the Fe oxide could be largely reduced, and hence, the growth of the Si oxide shell catalyzed by Fe oxide is greatly inhibited. Maybe because the oxygen contamination in the annealing gas is low, and most of the oxygen gas in the annealing tube has been expelled out by flowing forming gas through the tube for more than 20 min before annealing, the 10 % hydrogen in the annealing gas seems to be enough to react with the residual oxygen. Therefore, the formation of the Si oxide shell is much decreased. On the other hand, the decrease in the SiO_2_ shell thickness and increase in crystalline Si core diameter may result in the formation of straight Si NWs. According to the points as reported in references [39, 40], when the NWs mainly consist of amorphous oxide, there is no crystalline phase to stabilize the growth direction anymore, resulting in curved NWs. On the contrary, when the NWs mainly consist of no-oxide crystal, straight NWs would be preferentially formed. As a result, by annealing in 10 % H_2_ forming gas, straight Si NWs with thin oxide shells can be formed.

### Characteristic of Si NWs

**Fig. 3:**
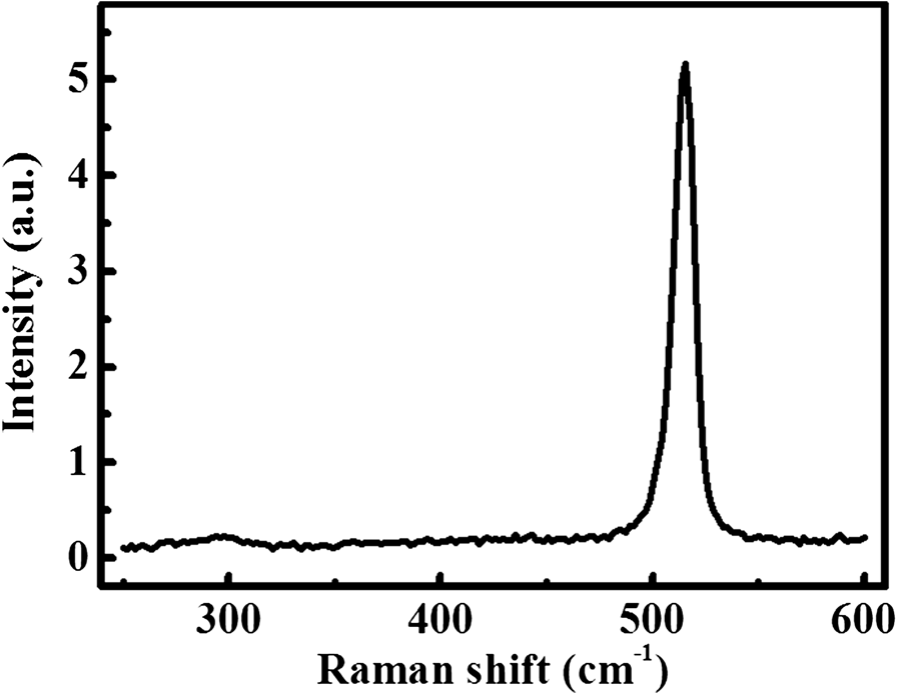
Raman spectrum of the Si NWs synthesized in 10 % H_2_ forming gas at 1250 °C. The NWs were scraped off the Si wafer and deposited on the Ta substrate to avoid the Raman signal from the Si substrate

The compositional and structural information of the Si NWs formed in 10 % H_2_ forming gas are further checked by Raman spectra and TEM measurements. The Raman spectrum of the NWs annealed at 1250 °C is shown in Fig. 3. From the Raman spectrum, a sharp peak located at about 516 cm^−1^ is observed, which exhibits an obvious redshift to the bulk Si peak at 520 cm^−1^, consistent with the results reported on Si NWs in previous literatures [41, 42]. The Raman spectra are repeatedly measured on different spots of the same sample or on different samples prepared at the same or different temperatures (1200~1300 °C). Similar Raman spectra are obtained, except the peak position varies from 506 to 518 cm^−1^ (not shown here). As the difference of the Raman peak shift can be attributed to the different diameters of the NWs [41, 42], i.e., the large Raman peak shift comes from the NWs with small diameters, the Raman results suggest that Si NWs with different diameters are formed under all these conditions. On the other hand, no broad bumps centered at 460 and 600 cm^−1^ are observed in the Raman spectra, which are corresponding to the synthesis of SiO_x_ NWs as observed on 5 % H_2_ forming gas annealed samples [12]. Therefore, it can be declared that the NWs prepared in 10 % H_2_ forming gas are mainly formed with Si, consistent with the above etching result.

**Fig. 4:**
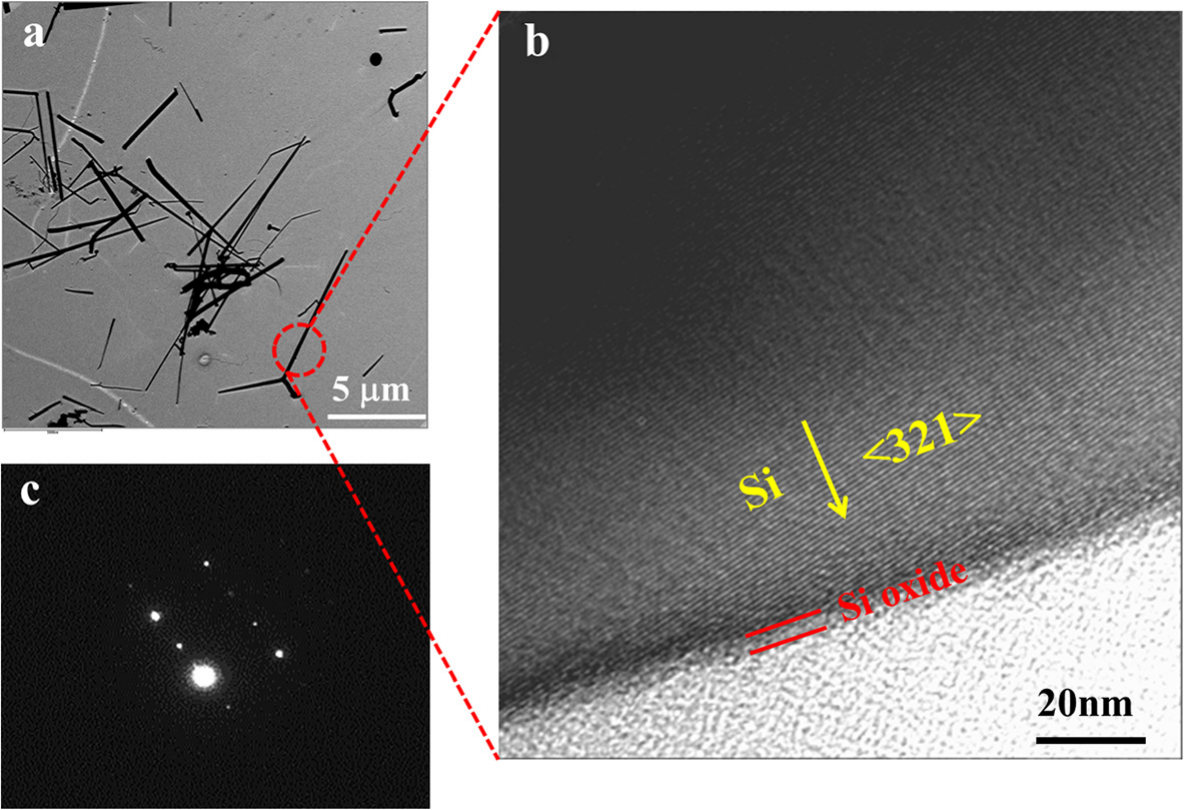
**a** TEM image of Si NWs deposited on the Cu mesh, which are synthesized in 10 % H_2_ forming gas at 1250 °C. **b** The HRTEM image and **c** the corresponding SAED pattern of the single NW labeled in **a**

The TEM images of the Si NWs prepared in 10 % H_2_ forming gas at 1250 °C and deposited on the Cu mesh are shown in Fig. 4. In the low-resolution TEM image (Fig. 4a), straight Si NWs with diameters varied from 40 to 200 nm are observed. Figure 4b presents the high-resolution image of the single NW as circled in Fig. 4a. From this image, it can be achieved that the NW is formed by crystal silicon, along the direction of <321>. At the outside of the NW, a thin bright layer can be observed, which should be attributed to the oxide layer. This oxide layer is only about 4 nm in thickness, consistent with the HF etching and Raman results. The selected area electron diffraction (SAED) image is presented in Fig. 4c, which well confirms the result obtained by TEM. From the above results, it can be concluded that the Si NWs fabricated at 1200~1300 °C in the 10 % H_2_ forming gas are mainly formed by crystal Si, covered with a very thin oxide shell.

### Electrical Measurements on Si NWs

**Fig. 5:**
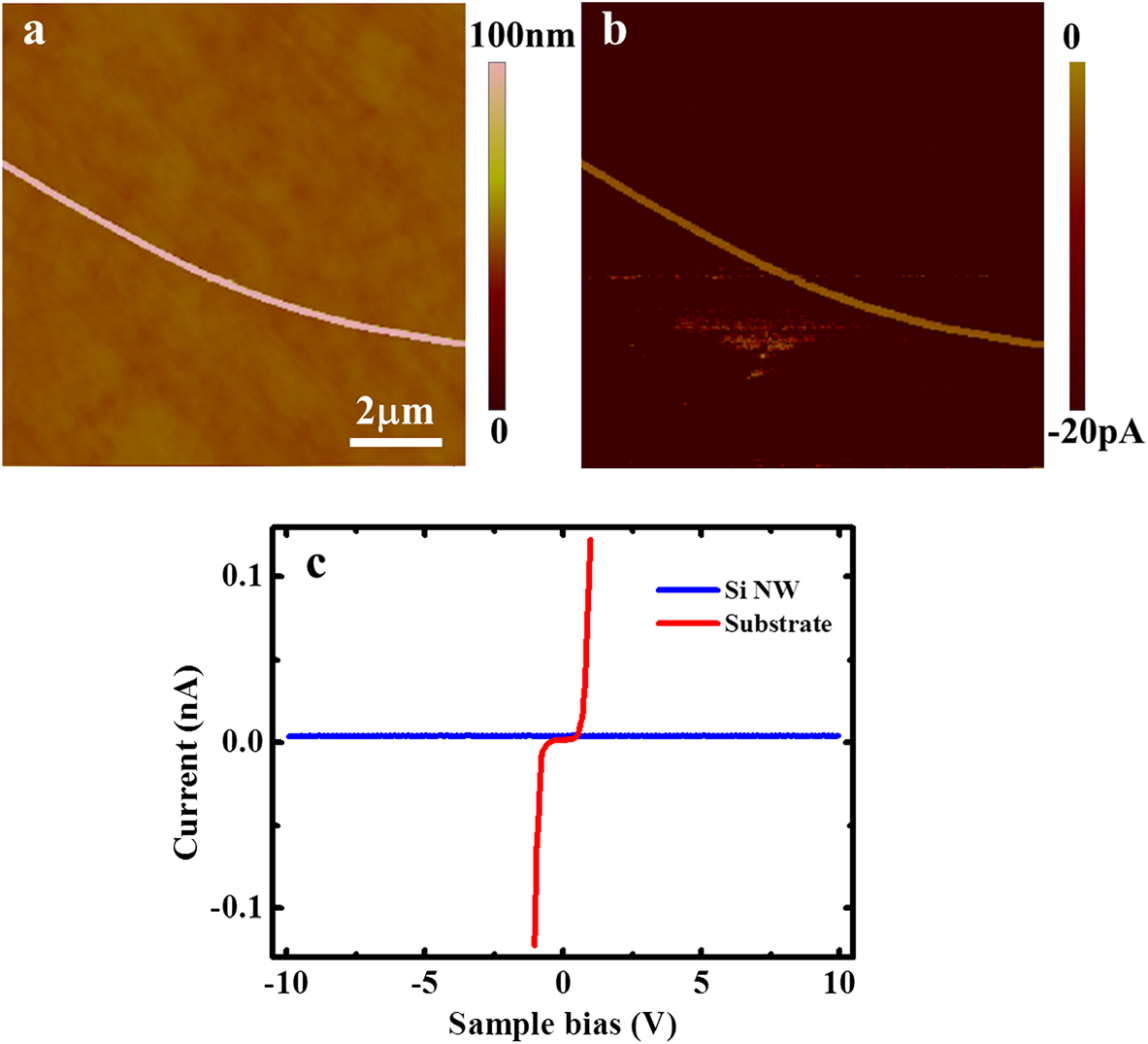
**a** Topography and **b** current image of single Si NWs deposited on the Si substrate measured by CAFM at a sample bias of −2 V. **c** Typical *I*-*V* characteristic curves measured on the Si NW and Si substrate

The conductive properties perpendicular to the NW are measured on the NWs deposited on a p-type Si substrate by CAFM. The topography and current images measured at −2 V are shown in Fig. 5a, b, respectively. It should be mentioned that the influence of Fe on the NWs’ electrical properties is not taken into consideration, as it has been demonstrated by previous TEM investigations that Fe should still be on or near the Si wafers and was rarely found in individual NWs prepared by the ultrasonic method [12]. From Fig. 5b, it can be seen that no current can be measured on the Si NW, whereas obvious current is detected on the Si substrate. The *I*-*V* curves measured on the NW and the substrate are presented in Fig. 5c. While the *I*-*V* curve measured on the substrate exhibits a good metal-semiconductor contact characteristic, that measured on the NW shows no detectable current up to ±10 V. We also deposit the Si NWs on the HOPG substrate as well as increase the normal force up to 300 nN, and again no current can be detected on the NW. The main problem may be due to the small contact area between the NW and the tip, as well as that between the NW and the substrate, resulting in huge contact resistances. As the conductance is not improved obviously when applied force increases and the Si NWs are spread on the substrate loosely, the contact between the NW and the substrate may be the dominating factor. Besides the huge contact resistances, the resistance of the NW itself is also large. It is because in CAFM, the measured resistance mainly comes from the top layer of the sample, since the resistance from the beneath layers decreases quickly with the current path due to the increased conductive area. However, for the cylindrical NWs, the conductive area is greatly restricted, which is especially small at the bottom of the NW also due to the small contact area between the NW and substrate, resulting in a large NW resistance. In addition, the native oxide layer exists unavoidably on both surfaces of the NW and substrate when the experiments are performed in ambient, which even lowers the current. As a consequence, no current can be detected on the NW by CAFM.

**Fig. 6:**
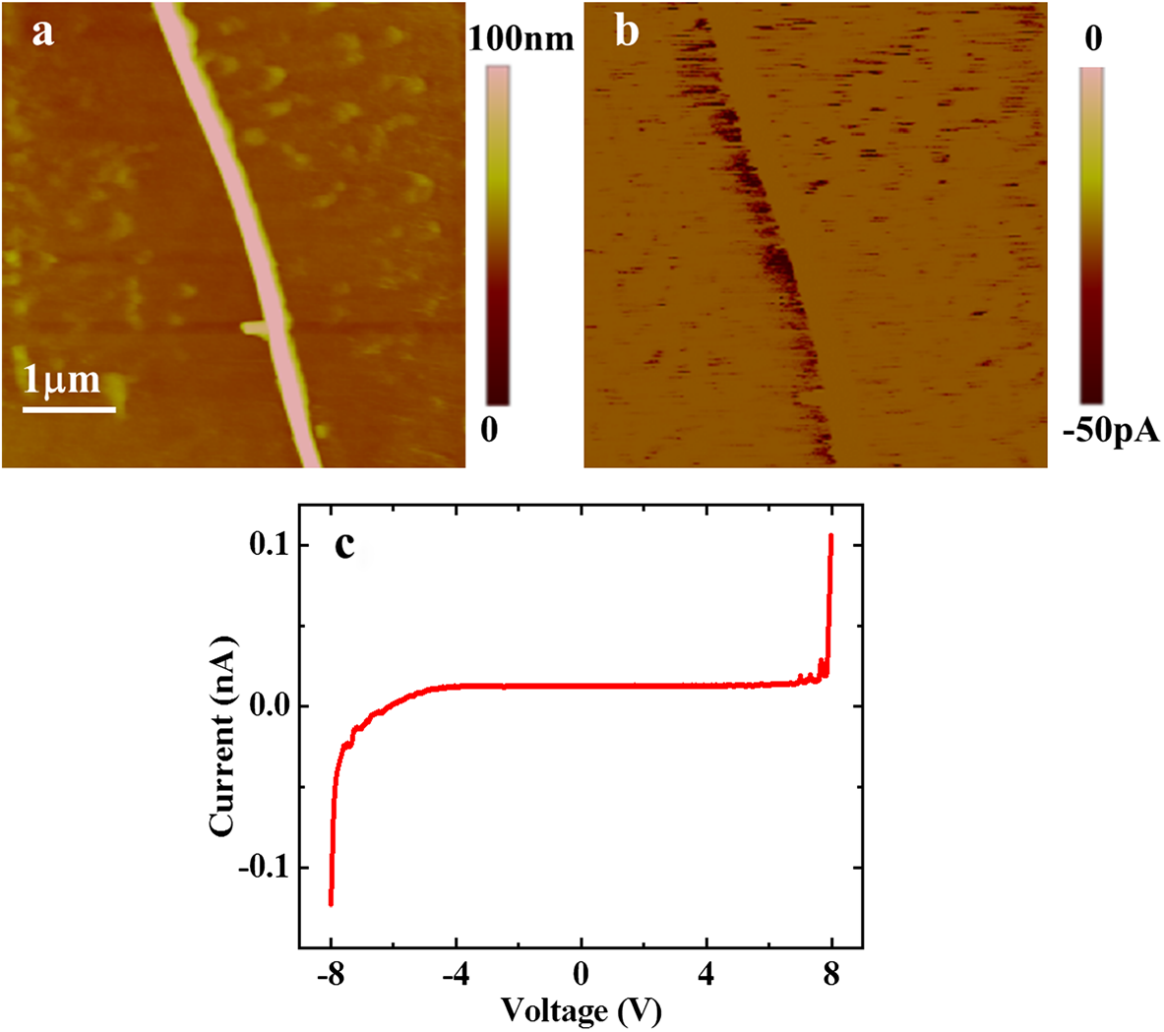
**a** Topography and **b** current image of single Si NWs deposited on the SiO_2_-covered Si substrate measured by CAFM at a sample bias of −5 V with the method as shown in Fig. 1a. **c** The typical *I*-*V* curve obtained on the Si NW

The above consideration is supported by the lateral conductive measurement along the NW. In this measurement, one side of the NWs is enwrapped by the Ag electrode, as shown in Fig. 1a, which can greatly increase the contact area between the NW and the electrode and decrease the contact resistance at this side correspondingly. Though the contact area between the tip and NW is still small, the total resistance with single small-area contact (tip-NW) should be much smaller than that in the perpendicular measurement with double small-area contacts. The topography and current images measured by CAFM at −5 V are shown in Fig. 6a, b, respectively. Now current, even still poor, can be measured at the edge of the NW, where the contact area is a little larger than other locations. In the current image obtained in the retract loop, the current at the other edge of the NW is measured. As the Ag electrode is located above the measured area, a slight decrease of current is detected along the NW from top to bottom. The *I*-*V* curve measured on the NW is plotted in Fig. 6c, which exhibits a typical metal-semiconductor contact characteristic. It again indicates that the Si NW is mainly formed by Si and the oxide layer is thin enough for electron or hole tunneling.

By comparing the conductance measurements perpendicular to and along the NW, it can be suggested that the poor measured conductance is mainly attributed to the electrical contact(s), since the current is largely increased when decreasing the number of small-area contacts from double to single. The results further suggest that the contact between the NW and the substrate should be the dominating factor, and current can be measured by improving this contact. On the other hand, the Si NWs exhibit typical semiconductive conductance themselves. So from all the above results, it can be demonstrated that straight, conductive, and crystalline Si NWs are fabricated by using forming gas with increased hydrogen ratio, which should have potential applications in nanoelectronics. Our results also suggest, after solving the contact problems, CAFM should be an effective approach to measure the conductive properties on single Si NW along the two directions without the need of nanoelectrode fabrications.

## Conclusions

By increasing the hydrogen ratio in the forming gas, straight and crystal Si NWs with a very thin oxide shell are fabricated. Both the conductive properties along and perpendicular to the NW are investigated by CAFM. Due to the poor electrical contact between the NW and the substrate as well as that between the NW and the tip, together with the native oxide layer, the conductive measurement should be further improved. Nevertheless, our results still present that the Si NWs exhibit typical semiconductive conductance, which may have potential applications in nanoelectronics.
